# Usefulness of nailfold videocapillaroscopy for systemic sclerosis

**DOI:** 10.1186/s41232-016-0001-x

**Published:** 2016-04-25

**Authors:** Satoshi Kubo, Yoshiya Tanaka

**Affiliations:** The First Department of Internal Medicine, School of Medicine, University of Occupational and Environmental Health, 1-1 Iseigaoka, Yahata-nishi-ku, Kitakyushu, Fukuoka 807-8555 Japan

**Keywords:** Systemic sclerosis, Nailfold videocapillaroscopy, Microvascular changes, Qualitative assessment, Semi-quantitative assessment

## Abstract

Systemic sclerosis is a complex disease that involves “autoimmunity,” “inflammation,” “fibrosis,” and “vasculopathy.” Microvascular damage and dysfunction particularly represent the earliest morphological and functional markers of systemic sclerosis. These morphological changes and progressions can be detected by nailfold videocapillaroscopy (NVC).

In 2013, the American College of Rheumatology and European League Against Rheumatism (ACR/EULAR) proposed a new set of criteria for systemic sclerosis for the first time in 30 years. Items are given a weighted score, and a score more than 9 indicates systemic sclerosis. These classification criteria encompass a broader spectrum of systemic sclerosis patients including those with early stage and with excellent sensitivity and specificity. Notably, nailfold capillary abnormalities were one of the new items in the criteria. Moreover, these abnormalities are also markers of systemic sclerosis severity and progression, as reduced capillary density has been associated with a high risk of developing digital skin ulcers and pulmonary arterial hypertension. Since microvascular damage and dysfunction represent early markers of systemic sclerosis, qualitative and semi-quantitative assessment of videocapillaroscopy images is expected in clinical application and treatment outcome assessment.

Despite the potential for targeted therapies in systemic sclerosis, there is no established therapy as yet. This may be due to several reasons. First, no fully validated outcome measures exist. Second, diagnosis of systemic sclerosis is often delayed and early intervention is difficult. Moreover, systemic sclerosis has clinical heterogeneity. Appropriate use of NVC helps to overcome these issues. Moreover, NVC may be useful in evaluating the pathogenesis of systemic sclerosis.

## Background

In 2013, the American College of Rheumatology and the European League Against Rheumatism (ACR/EULAR) collaborated to propose a new set of criteria for systemic sclerosis for the first time in 30 years [1, 2]. These criteria focused on the early diagnosis of systemic sclerosis. Seven items, namely, skin thickening of the fingers, fingertip lesions, telangiectasia, abnormal nailfold capillaries, pulmonary arterial hypertension and/or interstitial lung disease, Raynaud’s phenomenon, and systemic sclerosis-related autoantibodies, are given a weighted score; a score more than 9 indicates systemic sclerosis. These classification criteria encompass a broader spectrum of systemic sclerosis patients including those with early- and late-stage disease and with excellent sensitivity and specificity. The most significant change in these criteria was the inclusion of nailfold capillary abnormalities as one of the new items. It is now possible to diagnose systemic sclerosis with this scoring system before skin thickening of the fingers is detected.

## Role of NVC

Nailfold videocapillaroscopy (NVC) is the best and safest method to detect and analyze morphological microvascular abnormalities. NVC can detect normal capillary morphology in healthy individuals or those experiencing primary Raynaud phenomenon. However, abnormal capillaroscopic findings suggest the possibility of secondary Raynaud phenomenon [3]. Specific NVC changes known as the “systemic sclerosis pattern” are typical of the microvascular involvement in systemic sclerosis [4]. This review will summarize the “systemic sclerosis pattern,” which is widely accepted worldwide.

## Normal capillary morphology

**Fig. 1:**
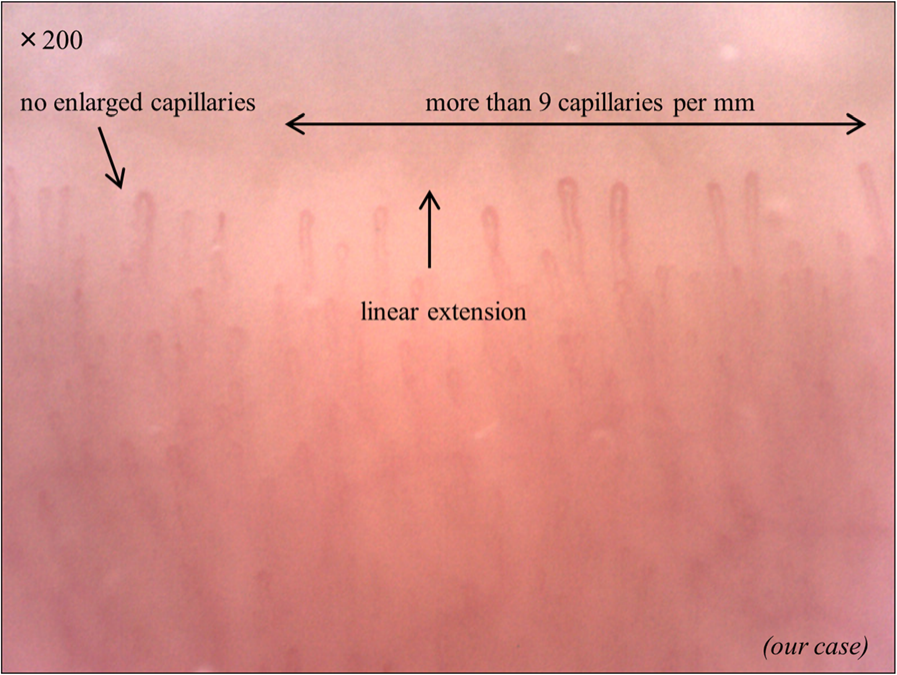
Representation of the nailfold videocapillaroscopy analysis of healthy donor. The vessels diameter is less than 20μm. Capillaries form a straight line toward distal. The number of capillaries is more than nine with in 1mm of the row of the nailfold bed

This section will address the normal morphology of capillaries in healthy individuals [5]. Healthy subjects have numerous thin and linear capillaries (Fig. 1).

Normal capillary morphology has several common characteristics. First, the diameter of normal vessels is less than 20 μm. However, it could be more than 20 μm in some capillaries in healthy individuals. However, the detection of even a single loop with a homogeneous increase to a diameter more than 50 μm should be considered a potential marker of microangiopathy. Second, capillaries are distributed with their major axis running parallel to the skin surface. Finally, there are more than 9 capillaries within 1 mm of the row of the nailfold bed. Abnormalities in these findings (fineness, direction, and number of capillaries) are seen in patients with systemic sclerosis. These sequential capillaroscopic changes are typical of the microvascular involvement in systemic sclerosis. Furthermore, quantitative and semi-qualitative scoring is possible for these findings [6].

## Usefulness of NVC for systemic sclerosis diagnosis

**Fig. 2:**
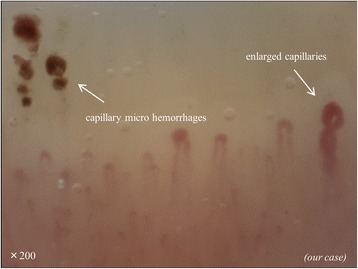
Representation of the nailfold videocapillaroscopy analysis of patients with systemic sclerosis. Enlarged capillaries and pericapillary hemorrhages at the nailfold are shown

The 1980 ACR classification criteria [7, 8] for systemic sclerosis lack sensitivity for the diagnosis of early systemic sclerosis. The new classification criteria encompass a broader spectrum of systemic sclerosis and focus on early diagnosis [1, 2]. Sensitivity and specificity for the new classification criteria were 91 and 92 %, respectively, while they were 75 and 72 %, respectively, for the 1980 ACR classification criteria. The new criteria include “abnormal nailfold capillaries,” which are enlarged capillaries and/or capillary loss with or without pericapillary hemorrhages at the nailfold (Fig. 2). Although capillaroscopy can be performed with highly specialized equipment such as in videocapillaroscopy, dermatoscopes (×30 magnifications) suffice for distinguishing between normal and abnormal nailfold capillaries. Therefore, there are no clear definitions of capillary thickness and number for the criteria. However, it is desirable for physicians caring for systemic sclerosis patients to use videocapillaroscopy (×200 magnifications) for an accurate measurement of capillary fineness and number.

## Qualitative and semi-quantitative scoring of capillary

Although enlarged capillaries, capillary loss, and pericapillary hemorrhages are included in the new criteria, the severities of these findings are not mentioned. Qualitative and semi-quantitative scoring of systemic sclerosis patterns by NVC has been introduced recently and validated [6, 9].

**Fig. 3:**
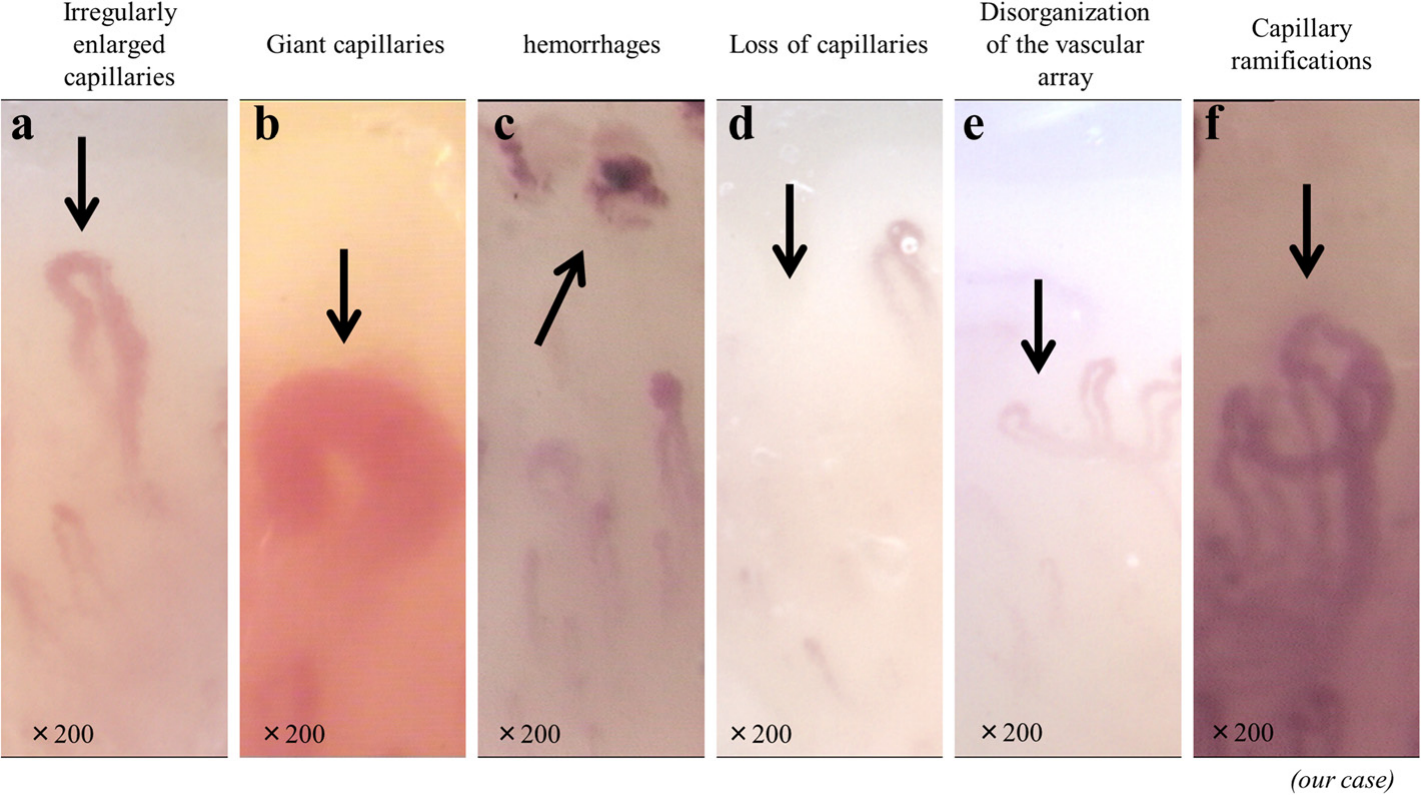
The six capillaroscopic parameters. **a** Irregularly enlarged capillary. **b** Giant capillary. **c** Micro hemorrhage. **d** Loss of capillaries. **e** Disorganization of the microvascular array. **f** Capillary ramifications

The following capillaroscopic parameters were scored: presence of enlarged and giant capillaries, hemorrhages, loss of capillaries, disorganization of the microvascular array, and capillary ramifications. The 6 parameters were defined as follows [6]: (a) irregularly enlarged capillaries (Fig. 3a) were defined as an increase in capillary diameter (homogeneous or irregular) >20 μm; (b) giant capillaries (Fig. 3b) as homogeneously enlarged loops with a diameter >50 μm; (c) microhemorrhages (Fig. 3c) as dark masses due to hemosiderin deposits; (d) loss of capillaries (Fig. 3d) was indicated by lower than normal capillary numbers (the normal range was adopted from literature; average 9 capillaries per linear mm at the distal row of the nailfold); (e) disorganization of the microvascular array (Fig. 3e) seen as irregular capillary distribution and orientation along with heterogeneity in the loop shape; and (f) capillary ramifications (Fig. 3f) such as branching, bushy, or coiled capillaries that often originated from a single normal sized capillary. Except for irregularly enlarged capillaries, these parameters characterize overt systemic sclerosis. Some habits such as manicures, onychophagia, and guitar playing cause microhemorrhages, which obviously do not depend on any pathological condition. Hence, patients should remove nail polish 2 weeks before the examination.

Qualitative and semi-quantitative assessment can be made by combining these findings. The NVC pattern is classified into three different patterns, namely, early, active, and late, which are clearly distinct from the normal pattern (Table 1). The early pattern is characterized by few giant capillaries, few capillary microhemorrhages, and no evident loss of capillaries. The active pattern comprises many giant capillaries, many capillary microhemorrhages, and moderate loss of capillaries. The late pattern is characterized by irregular enlargement of capillaries, almost no giant capillaries and microhemorrhages, severe loss of capillaries with extensive avascular areas, ramified capillaries, and intense disorganization of the normal capillary array. The natural progression of microvascular damage is from the early pattern to the late pattern [10]. The presence of anti-Scl70 antibodies (also called anti-topoisomerase I) seems to be related to earlier expression of the active and late pattern. On the other hand, anti-centromere antibody positivity seems to be related to delayed expression of the late NVC pattern [11].

**Table 1:** Nailfold videocapillaroscopy analysis of healthy donor

Early pattern	Active pattern	Late pattern
Giant capillaries <33 %	Giant capillaries >33 %	Giant capillaries <33 %
	Capillary microhemorrhage >33 %	Capillary microhemorrhage <33 %
No loss of capillaries	Loss of capillaries < 66 %	Loss of capillaries >66 %
No disorganization of the capillary array	Disorganization of the capillary array <66 %	Disorganization of the capillary array >66 %
No ramified capillaries	Ramified capillaries <33 %	Ramified capillaries >66 %

A semi-quantitative rating scale (score 0–3) should be adopted to score each of the aforementioned capillary abnormalities (0 = no changes, 1 = less than 33 % capillary alteration, 2 = 33–66 % capillary alteration, 3 = more than 66 % capillary alteration, per linear mm). The mean score value for each parameter should be calculated from the analysis of at least 2 mm in the center of the nailfold of each finger. The scores of irregularly enlarged capillaries, giant capillaries, and microhemorrhages are combined for the “A score.” Additionally, the scores for the loss of capillaries, disorganization of the microvascular array, and capillary ramifications are combined for the “B score.” The A score is higher in the early phase of systemic sclerosis, while B score is higher in the late phase. Interestingly, the A score decreases over time. In contrast, the B score increases significantly during the progression of microvascular damage [6]. We also observed this trend in our systemic sclerosis cohort (*n* = 89), and half of the patients showed progressive nailfold capillary changes. Moreover, the progression of nailfold capillary abnormalities in systemic sclerosis reflects organ involvement.

The earliest stage of microangiopathy is characterized by the appearance of giant capillaries. Concomitant microhemorrhages arise as a consequence of damaged microvessel walls. Subsequently, normally shaped capillaries progressively change, with all capillaries becoming irregularly enlarged and evident loss of capillaries. In the advanced stage of microangiopathy, both giant capillaries and microhemorrhages disappear, and neoangiogenesis with ramified capillaries induces microvascular array disorganization.

Several methods such as the Steinbrocker method (qualitative measure of radiographic damage) and the Sharp method (semi-quantitative measure of radiographic damage) have been introduced for scoring plain radiographs in rheumatoid arthritis patients to evaluate the disease course. Likewise, qualitative and semi-quantitative scoring of capillaries will become more important for the evaluation of the systemic sclerosis disease course.

## Clinical use of NVC

Multiple candidate therapies such as tocilizumab, rituximab, and autologous stem cell transplantation are being clinically evaluated in systemic sclerosis patients. Especially, tocilizumab is expected to be useful for treating systemic sclerosis. However, none of the targeted therapies has become an established therapy. There are several obstacles for clinical trials in systemic sclerosis. First, there are no fully validated outcome measures. Second, diagnosis of systemic sclerosis is often delayed and early intervention is difficult. Moreover, systemic sclerosis has clinical heterogeneity [12]. To date, early diagnosis of systemic sclerosis can only be made using the new classification criteria. On the other hand, a lack of validated outcome measures was identified in a systemic lupus erythematosus clinical trial. Thus, it is important to have validated outcome measures to obtain good clinical evidence. For example, biological disease-modifying anti-rheumatic drugs revolutionized the treatment of rheumatoid arthritis patients. One of the reasons for this revolution was that highly validated indices such as the simplified disease activity index and 28-joint disease activity scale were widely accepted worldwide for objectively evaluating disease activity, and a lot of clinical evidence was provided. As we have shown in this paper, NVC is useful for both early diagnosis and evaluation of disease progression. Scoring of capillary abnormalities has become very important because new treatments seem to be effective in modifying the capillary bed morphology in systemic sclerosis patients.

## Conclusions

Systemic sclerosis is a complex disease involving “autoimmunity,” “inflammation,” “fibrosis,” and “vasculopathy.” Therapeutic goals in systemic sclerosis include minimization of damage from early inflammation and autoimmunity, restoration of vascular homeostasis, promotion of structural connective tissue repair, and resolution of scarring [12]. As in other autoimmune diseases, early diagnosis and intervention are important for achieving these goals. NVC has the potential to provide valuable information for early diagnosis, disease activity, disease progression, qualitative assessment, and pathogenesis of systemic sclerosis. Further clinical application of NVC and its epidemiology are required.
